# Identification of FOS as a Candidate Risk Gene for Liver Cancer by Integrated Bioinformatic Analysis

**DOI:** 10.1155/2020/6784138

**Published:** 2020-03-22

**Authors:** Jin-Wu Hu, Guang-Yu Ding, Pei-Yao Fu, Wei-Guo Tang, Qi-Man Sun, Xiao-Dong Zhu, Ying-Hao Shen, Jian Zhou, Jia Fan, Hui-Chuan Sun, Cheng Huang

**Affiliations:** ^1^Department of Liver Surgery and Transplantation, Zhongshan Hospital, Fudan University, Key Laboratory of Carcinogenesis and Cancer Invasion (Fudan University), Ministry of Education, Shanghai 200032, China; ^2^Department of General Surgery, Shanghai Tenth People's Hospital Affiliated with Tongji University, 301 Yanchang Road, Shanghai 200072, China; ^3^Department of General Surgery, Institute of Fudan-Minhang Academic Health System, Minhang Hospital, Fudan University, Shanghai 201199, China

## Abstract

Liver cancer is a lethal disease that is associated with poor prognosis. In order to identify the functionally important genes associated with liver cancer that may reveal novel therapeutic avenues, we performed integrated analysis to profile miRNA and mRNA expression levels for liver tumors compared to normal samples in The Cancer Genome Atlas (TCGA) database. We identified 405 differentially expressed genes and 233 differentially expressed miRNAs in tumor samples compared with controls. In addition, we also performed the pathway analysis and found that mitogen-activated protein kinases (MAPKs) and G-protein coupled receptor (GPCR) pathway were two of the top significant pathway nodes dysregulated in liver cancer. Furthermore, by examining these signaling networks, we discovered that FOS (Fos proto-oncogene, AP-1 transcription factor subunit), LAMC2 (laminin subunit gamma 2), and CALML3 (calmodulin like 3) were the most significant gene nodes with high degrees involved in liver cancer. The expression and disease prediction accuracy of FOS, LAMC2, CALML3, and their interacting miRNAs were further performed using a HCC cohort. Finally, we investigated the prognostic significance of FOS in another HCC cohort. Patients with higher FOS expression displayed significantly shorter time to recurrence (TTR) and overall survival (OS) compared with patients with lower expression. Collectively, our study demonstrates that FOS is a potential prognostic marker for liver cancer that may reveal a novel therapeutic avenue in this lethal disease.

## 1. Introduction

Liver cancer is a lethal disease often caused by liver damage associated with virus infection, excessive alcohol, or other liver disease [1]. It ranks the sixth most common cancer worldwide and accounts for the fourth most common cause of cancer related death due to lack of effective therapies [2]. Currently, the most curative therapy for liver cancer is surgery, but most patients are not suitable for this treatment because of the advanced tumor stage at the time of diagnosis. Currently, there are limited targeted therapy agents or drug combinations that can extend the survival time of these patients [3].

In recent years, the studies of cancer genetics and genomic sequencing have contributed to deeper understanding of the underlying cause of liver cancer [4]. The gene expression profiling analysis has improved the classification of liver cancer subtypes [5]. The discovery of mutations in BRAF (B-Raf proto-oncogene, a serine/threonine kinase) and EGFR (epidermal growth factor receptor) by DNA sequencing contributed to the development of new targeted therapies for liver cancer patients carrying these mutations [6, 7]. Based on gene expression patterns of liver cancer, MST1 (or so called “hepatocyte growth factor-like protein”) was found to be lowly expressed in liver cancer sample induced by p53 [8], and its role in regulation of hepatocyte differentiation has been studied previously [9]. In addition, miR-122 was found to be expressed at lower levels in liver cancer cells compared to normal hepatocytes by gene expression profiling, thus contributing to its function on liver cancer cell migration and invasion [10]. miR-122 has been suggested as a diagnostic and prognostic marker for liver cancer. However, the molecule marker for liver cancer has not been fully investigated.

In our current study, we aimed to identify novel genes or pathways that may be important for liver cancer progression by performing integrated analyses of mRNA and miRNA in liver cancer patients. The global genomic analysis may be helpful to discover new cancer-associated genes and reveal the molecular basis important for liver cancer development.

## 2. Materials and Methods

### 2.1. Data Acquisition and Differential Expression Analysis

The miRNA and mRNA expression datasets of liver cancer were downloaded from TCGA database (https://gdc-portal.nci.nih.gov/). There were 372 liver cancer tissues and 50 tumor-adjacent tissues for miRNA expression profiles and 371 tumor samples and 50 adjacent tissues for mRNA expression dataset.

Compared with the tumor-adjacent tissues, the differentially expressed (DE) mRNAs (genes) and DE miRNAs in tumor tissues were identified by edgeR package in R. Corrected *P* value <0.05 and |log_2_FC(fold change)| >1.0 were considered as significant.

### 2.2. Risk Genes for Liver Cancer Analysis

The targets of experiment-validated DE miRNAs were predicted based on miRecords, miRTarBase, and TarBase. The target genes were mapped to DE mRNAs, and the overlapped genes were defined as the risk genes of liver cancer.

### 2.3. Differentially Expressed Pathway Analysis

KEGG (Kyoto Encyclopedia of Genes and Genomes, http://www.genome.jp/kegg/pathway.htm) is a database associated with gene function annotation, which is comprised of four main databases including GENES database, PATHWAY database, LIGAND database, and BRITE database [11]. The PATHWAY database is the collection of pathways for various biological processes. All the human pathways and their related genes were downloaded from the PATHWAY database. The pathway expression value in each sample was calculated based on the median expression level of each gene involved in pathway. The formula was listed as follows:

(1) $$\begin{array}{c} {\text{Path}}_{ik}={\text{Median}}_{k}\left(g_{1},g_{2,}g_{3},...,g_{n}\right), \end{array}$$

where Path_*ik*_ represents the expression level of pathway *i* in sample *k* and *g*_1−*n*_ represents the expression value of each gene in pathway *i*.

Compared with controls, the differentially expressed pathways with corrected *P* value <0.05 and |log_2_FC| ≥ 1 in tumor samples were analyzed by edge package in R. Bidirectional clustering analysis of the differentially expressed pathways was carried out by pheatmap package in R.

### 2.4. The Correlated Pathway Pairs

The correlation between pathways in tumor and control samples was analyzed by Pearson correlation coefficient as follows:

(2) $$\begin{array}{c} P_{X,Y}=\frac{\sum \left(X-\bar{X}\right)\left(Y-\bar{Y}\right)}{\sqrt{\sum {\left(X-\bar{X}\right)}^{2}\sum {\left(Y-\bar{Y}\right)}^{2}}}, \end{array}$$

where P_*X*,*Y*_ represents the expression correlation coefficient between pathway *X* and Y and $\;\bar{X}$ and $\bar{Y}$ represent the mean expression value of pathway *X* and Y, respectively.

The pathway pairs with |correlation coefficient| >0.8 in both tumor and control samples were collected.

### 2.5. miRNA-Gene-Pathway Network Construction

In order to obtain risk gene-pathway pairs, the common risk genes in both pathways of correlated pathway pair were collected. miRNA-gene-pathway network was constructed based on risk gene-pathway pair and miRNA-risk gene interactions. Subsequently, the properties of network topology, such as degree, average shortest path length, betweenness centrality, closeness centrality, clustering coefficient, and topological coefficient, were analyzed by “network analysis” function of Cytoscape software. The disease classification accuracy of significant gene and miRNAs was predicted by ten-fold cross validation.

### 2.6. Quantitative Reverse Transcription-Polymerase Chain Reaction (qRT-PCR)

Total RNA was extracted using RNeasy mini kit (Qiagen) according to the manufacturer's protocols. Target genes were quantified using the SuperScript III Platinum SYBR green one-step qRT-PCR kit (Thermo Fisher Scientific, Inc.). GAPDH was used as an endogenous control, and the primer sequences used in this study were listed as follows: FOS, F: 5′-CCGGGGATAGCCTCTCTTACT-3′ R: 5′-CCAGGTCCGTGCAGAAGTC-3′; CALML3, F: 5′-CTTCTCCCTGTTTGACAAGGAT-3′ R: 5′-GTCGATCTCACTCATCATGTCC-3′; LAMC2, F: 5′-GACAAACTGGTAATGGATTCCGC-3′ R: 5′-TTCTCTGTGCCGGTAAAAGCC-3′; GAPDH, F: 5′-ATGGGGAAGGTGAAGGT-3′ R: 5′-AAGCTTCCCGTTCTCAG-3′. The hsa-miR-221 (assay ID000524; Thermo Fisher Scientific, Inc.), hsa-miR-222 (assay ID000525; Thermo Fisher Scientific, Inc.), hsa-miR-199b (assay ID000500; Thermo Fisher Scientific, Inc.), and hsa-mir-765 (assay ID002643; Thermo Fisher Scientific, Inc.) expression levels were determined using TaqMan MicroRNA Assays.

### 2.7. Patients and Follow-Up

382 patients who were diagnosed with HCC and underwent surgery from January to December in 2011 in Zhongshan Hospital were recruited. The inclusion criteria were as follows: (a) no prior cancer treatment, (b) pathologically diagnosed HCC, with complete resection of all tumor nodules with margins confirmed free of cancer by histologic examination, and (c) availability of complete clinicopathologic and follow-up data. The Barcelona Clinic Liver Cancer (BCLC) staging system was used to assess tumor stage. Tumor differentiation was determined according to the Edmondson grading system. Approval for research protocol and use of human subjects was obtained from the research ethics committee of Zhongshan Hospital. Informed consent was obtained from each patient. Follow-up ended in August 2017. Time to recurrence (TTR) was defined as the interval between surgery and the diagnosis of any type of recurrence including intra or extrahepatic recurrence identified by MR or CT. OS was defined as the interval between treatment and death of any cause or the last observation date [12].

### 2.8. Tissue Microarray (TMA) and Immunohistochemistry (IHC)

Immunohistochemical staining was performed using the avidin-biotin-peroxidase complex method. Primary anti-human-FOS antibodies were added to the slides and incubated at 4°C overnight, followed by rehydration and microwave antigen retrieval. Subsequently, secondary antibody was incubated at 37°C for 30 minutes. Slides were stained with 3′3-diaminobenzidine tetra hydrochloride and counterstained with Mayer's hematoxylin. The assessment of immunohistochemical staining was performed by two independent pathologists, and discrepancies were resolved by consensus. The intensity of FOS staining was stratified as weak or strong.

### 2.9. Statistical Analysis

Statistical analyses were performed using SPSS 20.0 software (IBM, Armonk, NY, USA). Experimental values for continuous variables are expressed as the mean ± standard error of the mean. Chi-squared tests, Fisher's exact probability tests, and Student's *t*-tests were used to evaluate the significance of differences between groups. If variances within groups were not homogeneous, a nonparametric Mann–Whitney test or Wilcoxon signed-rank test was used. The relationships between FOS expression and TTR or OS were analyzed using Kaplan–Meier survival curves and log-rank tests, respectively. *P* < 0.05 was considered statistically significant [13].

## 3. Results

### 3.1. Identification of Potentially Functionally Important mRNAs and miRNAs for Liver Cancer

In order to examine the potentially important mRNAs and miRNAs in liver carcinogenesis, first we performed analysis of differentially expressed (DE) mRNAs or miRNAs from liver cancer patients compared to normal tissues in TCGA database by using corrected-*P* value < 0.05 and |log_2_FC| > 1.0; a total of 405 DE genes (273 upregulated ones and 132 downregulated ones) were identified based on gene expression profiling of liver tumors compared to normals. In addition, 233 DE miRNAs (39 upregulated miRNAs and 194 downregulated miRNAs) were obtained based on the miRNA expression profiling in the same dataset.

**Figure 1:**
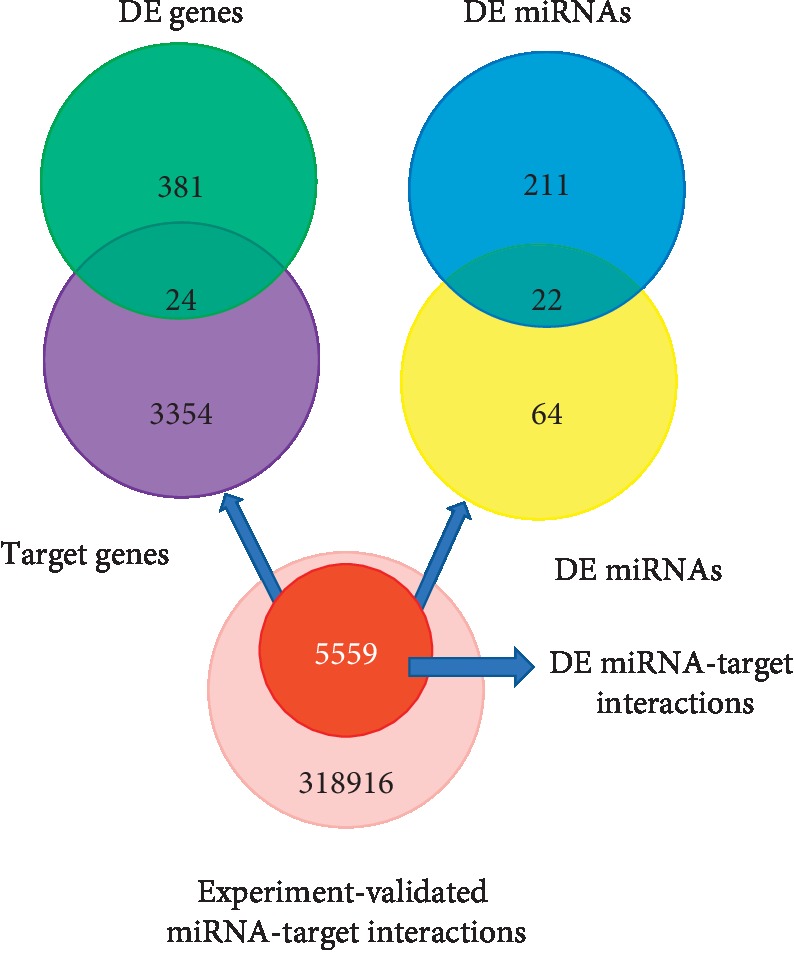
Identification of potentially functionally important mRNAs and miRNAs for liver cancer.

Next, to examine the functional network between miRNAs and their target genes, we obtained a total of 324475 experiment-validated miRNA-target interactions, among which there were 5559 DE miRNA-target interactions, including 86 DE miRNAs and 3378 target genes. After the target genes were mapped to DE genes, 35 DE miRNA-DE gene pairs were obtained, which included 22 DE miRNAs and 24 risk genes (Figure 1). This is a reasonably focused miRNA and gene list that will be used for our subsequent analysis in liver cancer.

### 3.2. Examination of Potentially Important Pathways for Liver Carcinogenesis

To determine the pathways dysregulated in liver cancer due to these differentially expressed mRNAs or miRNAs, we used the cutoff value of corrected *P* value < 0.05 and |log_2_FC| ≥ 1 to perform the gene set enrichment analysis with the KEGG pathway database. A total of 622 pathways were found to be significantly dysregulated in liver tumor samples compared to normal, which was presented as a heatmap plot in Supplementary [Supplementary-material supplementary-material-1]. Therefore, these pathways might have significant roles in liver tumorigenesis.

### 3.3. Elucidation of miRNA-Gene-Pathway Network in Liver Cancer

**Figure 2:**
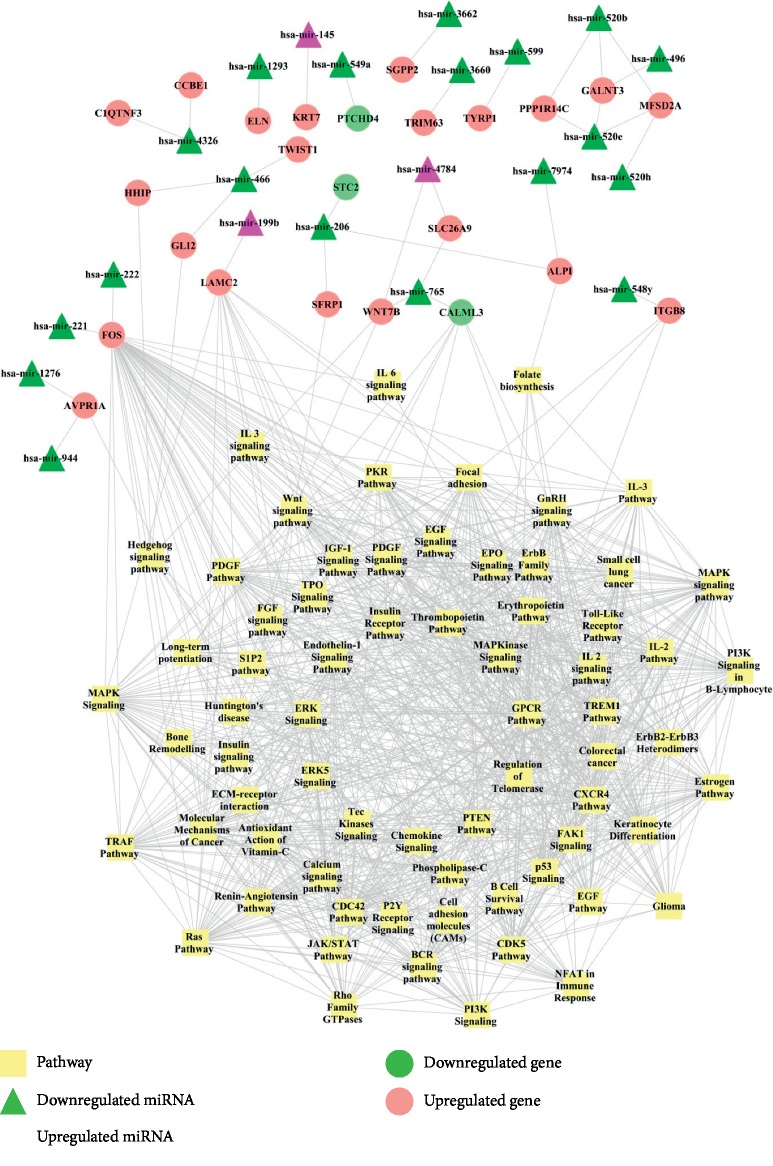
Elucidation of miRNA-mRNA-pathway network in liver cancer.

To examine the crosstalk between miRNA-mRNA dysregulated in liver cancer, which may yield more confidence on studying important oncogenic pathways in this cancer, we examined the miRNA-mRNA-pathway correlation. By using the |correlation coefficient| >0.8, we obtained 13424 correlated pathway pairs. After comparing the risk genes and pathway related genes, the miRNA-risk gene-pathway pair network was constructed, which was comprised of 1024 edges connecting 115 nodes (Figure 2).

**Figure 3:**
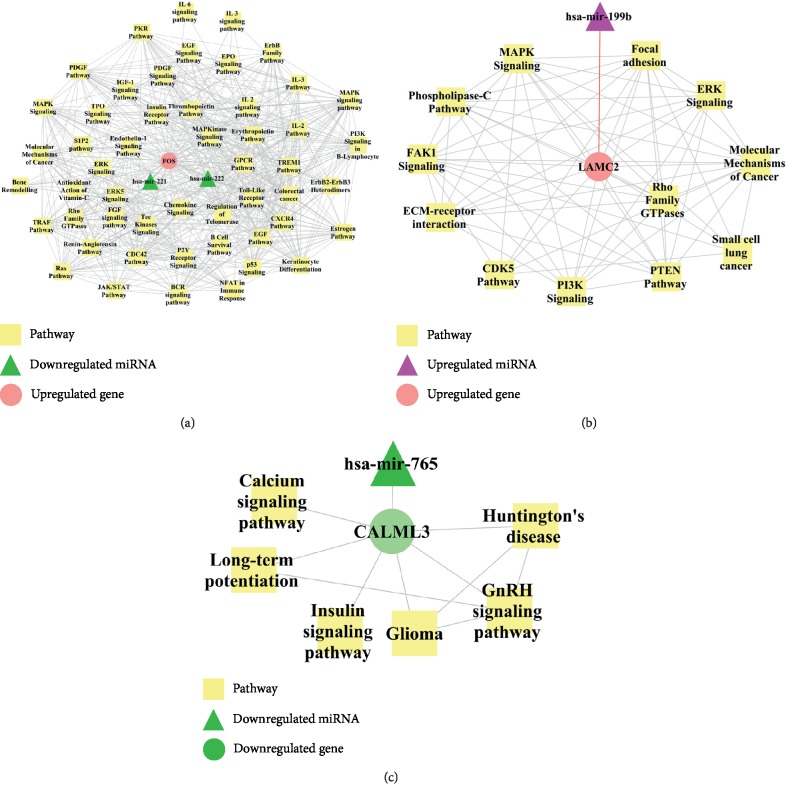
FOS, LAMC2, and CALML3 are three important genes involved in liver cancer by miRNA-mRNA-pathway network analysis.

Then, the topological properties of nodes in network were achieved by Cytoscape software. MAPK signaling pathway (degree = 53) and GPCR Pathway (degree = 51) were two of the most significant pathways in miRNA-gene-pathway network that showed most interactions with risk genes (Table 1). Further analyses of important genes in these pathways revealed that FOS (Fos proto-oncogene, AP-1 transcription factor subunit, degree = 54), LAMC2 (laminin subunit gamma 2, degree = 13), and CALML3 (calmodulin like 3, degree = 7) were the most significant gene nodes with high degrees indicating the pivotal role of these genes involved in liver cancer (Table 2). FOS was previously reported to be regulated by hsa-miR-221 and hsa-miR-222 and showed interactions with MAPK signaling pathway, JAK/STAT pathway, GPCR pathway, and insulin receptor pathway (Figure 3(a)). LAMC2 interacted with hsa-miR-199b and was involved in the pathways such as MAPK signaling, ERK signaling, PI3K signaling, and PTEN pathway (Figure 3(b)). CALML3 interacted with hsa-miR-765 and played a regulatory role in GnRH signaling pathway, long-term potentiation, and Huntington's disease (Figure 3(c)).

### 3.4. Validation of Significant Genes and Interacting miRNAs in Liver Cancer

**Figure 4:**
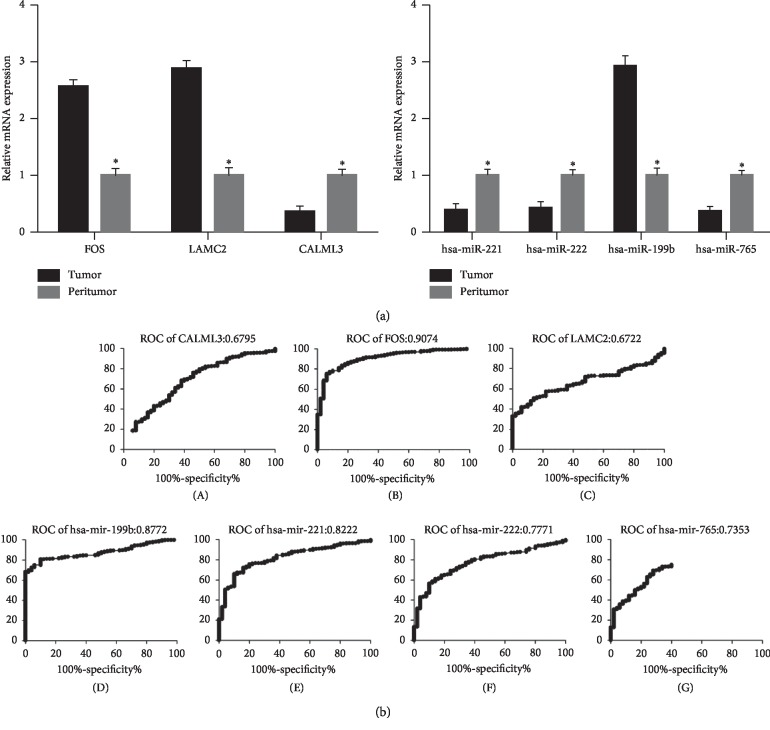
Clinical significance of FOS, LAMC2, and CALML3 with high degree and their related miRNAs.

To validate our miRNA-mRNA-pathway interaction network analysis, we examined the expression of FOS, LAMC2, CALML3, and their interacting miRNAs using 20 pairs of hepatocellular carcinoma samples and adjacent normal tissues by RT-PCR. Our results were highly consistent with our analysis from TCGA database (Figure 4(a)). Furthermore, the disease prediction accuracy of FOS, LAMC2, CALML3, and their interacting miRNAs was analyzed. As shown in Figure 4(b), FOS (0.9074), hsa-miR-199b (0.8772), and hsa-miR-221 (0.8222) displayed high receiver operating characteristic (ROC) values, among which FOS exhibited the highest prediction accuracy.

### 3.5. High FOS Expression Predicts Poor Prognosis in HCC Patients

**Figure 5:**
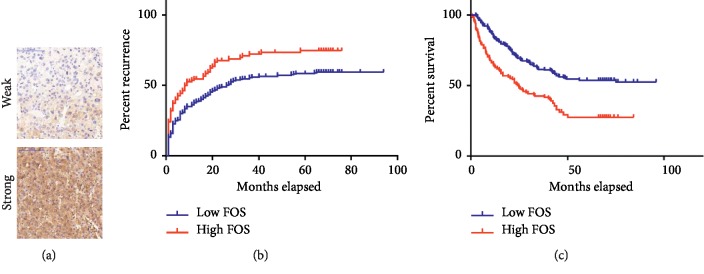
FOS was correlated with poor prognosis in HCC patients.

Based on our analysis above, we were motivated to study the relationship of FOS expression with the prognosis of HCC. To this end, a TMA containing 382 patients who underwent curative resection was immunostained with the previously characterized antibody against FOS. Basic pathological and clinical information for these 382 HCC patients enrolled is described in Table 3. These HCC patients were divided into two groups according to the FOS expression level (high or low) determined by the IHC staining intensity (strong or weak) (Figure 5(a)). Our statistical analysis showed that high expression of FOS was associated with Hepatitis B Virus (HBV) infection background, alpha-fetoprotein (AFP) level, and macrovascular invasion. Kaplan–Meier analysis revealed significantly shorter median overall survival (OS) in patients with higher FOS expression compared with those patients with lower FOS expression (24.7 months vs. not reached, ^*∗∗*^*P* < 0.01, Figure 5(b)). Similarly, patients with higher FOS expression displayed significantly shorter time to recurrence (TTR) compared with patients with lower expression (9 months vs. 26 months, ^*∗∗*^*P* < 0.01, Figure 5(c)).

Univariate and multivariate analysis showed that OS is correlated with FOS expression status (HR = 0.69 [0.60, 0.80], ^*∗∗*^*P* < 0.01, Table 4), HBsAg status, preoperative AFP, tumor size, and tumor encapsulation while TTR correlated with FOS expression status (HR = 0.79 [0.68, 0.90], ^*∗∗*^*P* < 0.01, Table 5), HBsAg status, tumor number, tumor size, and Edmondson stage (all *P* < 0.05).

Collectively, our results suggest that FOS can be used as an effective marker of prognosis in HCC.

## 4. Discussion

Tumor initiation is attributed to the process of genetic and epigenetic alterations of patients, which lead to gene expression alternations involved in tumor evolution. In this paper, we analyzed the gene expression and miRNA expression profiling of liver cancer by performing integrated bioinformatics analysis. The DE mRNAs and DE miRNAs were identified by comparing tumor tissues and controls followed by the miRNA-mRNA-pathway network construction. The integrative analyses of miRNA-mRNA-pathway network for identifying functionally relevant genes in liver cancer progression are not frequently used. By performing these new analyses, we attempted to pinpoint driver genes that may contribute to the development of liver cancer.

Our data showed that MAPK and GPCR pathways were two of the most significant nodes in miRNA-gene-pathway network. MAPKs are the family of serine-threonine kinases, which contain extracellular signal-regulated kinase (ERK), p38, and c-Jun NH2-terminal kinase (JNK). MAPK signaling pathway is activated by extracellular and intracellular stimuli and plays a key role in cell proliferation, differentiation, and other cellular activities [14]. MAPK signaling pathway has been reported to be implicated in pathogenesis of many different cancers. The dysregulation of MAPK signaling pathway in cancer development is frequently due to the mutations of signaling components involved in the pathway [15]. It is reported that the expression and activity of MAPK are significantly upregulated in primary liver cancer [16]. MAPK is found to be overexpressed in liver cancer patients and plays a key role in the growth and survival of liver cancer cells [17]. Targeting MAPK signaling pathway has been suggested to be one of the attractive candidates for cancer therapy. In the previous study, RAS/MAPK signaling pathway activity is reported to be upregulated in medulloblastoma based on gene expression profiling [18]. MAPK signaling pathway has been found to be the most important signaling node based on lncRNA profiling in breast cancer [19]. Consistent with these findings in breast cancer, our study also suggests the significance of MAPK signaling in liver cancer.

GPCRs are the family of seven-transmembrane receptors and play key roles in signal transmission involved in various physiological functions. GPCRs are overexpressed in various cancers and play key roles in tumor cell proliferation. Therefore, the dysfunction of GPCRs has been proven to promote the progression and metastasis of cancers [20]. For example, GPCRs have been reported to be involved in prostate cancer development [21]. However, the direct evidence for the role of GPCR pathway in liver cancer is lacking. There were some limited literatures suggesting that GPCRs are closely related to the activation of Hippo pathway [22] involved in liver growth and tumor formation [23]. All these literatures above suggest the significant roles of MAPK and GPCR pathways in liver cancer progression.

In our study, our data suggest that FOS is an important gene in signaling nodes involved in the interaction of MAPK and GPCR pathways. It is reported that c-FOS plays a key role in the signal transduction pathway which acts as a trans-activating as well as trans-repressing molecule [24]. The c-FOS is a mitogen responsive gene associated with cell proliferation. It is reported that c-FOS gene expression is activated by the combined effect of extracellular nucleotides and growth factors, which leads to increased calcium levels and proliferation in breast cancer cells [24]. In addition, the stimulation of c-FOS gene expression is implicated in activation of ERK signaling. Furthermore, overexpression of c-FOS gene is mediated by the G protein-coupled receptor GPR30 through E2 (17*β*-estradiol) and phytoestrogens in breast cancer cells [25]. Thus, both ERK signaling and GPR30 signaling are implicated in cell proliferation of breast cancer induced by FOS gene. The significant role of FOS gene is also identified in other cancers, such as bladder cancer [26], lung cancer [27], and colon cancer [28]. However, the role of FOS gene in liver cancer remains largely unclear. In our present study, FOS gene exhibits the highest prediction accuracy of liver cancer among the significant nodes in network, which suggests the potential diagnostic and therapeutic implication of FOS in liver cancer.

## 5. Conclusion

In conclusion, the co-activation of MAPK and GPCR pathways may be involved in liver cancer progression. FOS may be the risk gene for liver cancer by playing important roles in MAPK and GPCR pathways. FOS could be considered to be a candidate gene for diagnosis and therapy for liver cancer.

## Figures and Tables

**Table 1:** Top 15 pathways with high degree in miRNA-gene-pathway network.

Pathway	Degree	Average shortest path length	Betweenness centrality	Closeness centrality	Clustering coefficient	Topological coefficient
MAPK signaling pathway	53	1.670213	0.055032	0.598726	0.530479	0.40363
GPCR pathway	51	1.712766	0.030236	0.583851	0.576471	0.428607
Colorectal cancer	49	1.765957	0.044379	0.566265	0.544218	0.39325
CXCR4 pathway	47	1.787234	0.027533	0.559524	0.582794	0.407256
Molecular mechanisms of cancer	46	1.723404	0.040792	0.580247	0.578744	0.413043
MAPKinase signaling pathway	45	1.808511	0.020999	0.552941	0.625253	0.422222
CDC42 pathway	45	1.776596	0.018781	0.562874	0.621212	0.443305
PI3K signaling in B-Lymphocyte	44	1.840426	0.010117	0.543353	0.660677	0.445691
FAK1 signaling	42	1.840426	0.015468	0.543353	0.645761	0.465079
MAPK signaling	42	1.765957	0.02427	0.566265	0.644599	0.436607
Focal adhesion	41	1.840426	0.045292	0.543353	0.635366	0.456033
Estrogen pathway	41	1.819149	0.01352	0.549708	0.659756	0.454972
CDK5 pathway	41	1.861702	0.024969	0.537143	0.647561	0.431739
GnRH signaling pathway	40	1.882979	0.018458	0.531073	0.703846	0.461842
PDGF pathway	39	1.893617	0.005126	0.52809	0.746289	0.468864

**Table 2:** Top 10 genes with high degree in miRNA-gene-pathway network.

Gene symbol	Expression	Degree	Average shortest path length	Betweenness centrality	Closeness centrality	Clustering coefficient	Topological coefficient
FOS	Up_gene	54	1.787234	0.138354	0.559524	0.378756	0.3768
LAMC2	Up_gene	13	2.308511	0.021807	0.43318	0.692308	0.485577
CALML3	Down_gene	7	2.489362	0.032014	0.401709	0.190476	0.34026
WNT7B	Up_gene	4	2.62766	0.039645	0.380567	0	0.255952
ITGB8	Up_gene	4	2.723404	0.021353	0.367188	0.166667	0.380435
ALPI	Up_gene	3	3.361702	0.025814	0.297468	0	0.333333
AVPR1A	Up_gene	3	3.276596	0.042324	0.305195	0	0.333333
MFSD2A	Up_gene	3	1.666667	0.355556	0.6	0	0.666667
GALNT3	Up_gene	3	1.666667	0.355556	0.6	0	0.666667
SFRP1	Up_gene	2	2.765957	0.036954	0.361538	0	0.5

Up and down represent upregulated expression and downregulated expression, respectively.

**Table 3:** Clinical characteristics of HCC patients.

Clinical and pathologic indexes		High FOS	Low FOS
		*N* = 264	*N* = 118	*P*
Age, y	>50	172	73	0.56
	≤50	92	45	

Gender	Female	29	16	0.49
	Male	235	102	

HBsAg	Negative	55	36	0.05
	Positive	209	82	

With liver cirrhosis	No	6	1	0.44
	Yes	258	117	

Portal lymph node	Negative	255	112	0.41
	Positive	9	6	

AFP (ng/ml)	≤400	99	59	0.02^*∗*^
	>400	165	59	

MVI	Negative	200	83	0.31
	Positive	64	35	

Tumor number	Multiple	67	27	0.70
	Single	197	91	

Tumor size, cm	>5	146	69	0.58
	≤5	118	49	

PVTT	Negative	165	57	0.01^*∗*^
	Positive	99	61	

GGT (U/L)	>54	185	79	0.55
	≤54	79	39	

Tumor encapsulation	Complete	132	57	0.83
	None	132	61	

Edmondson stage	I-II	193	76	0.09
	II-IV	71	42	

*Note.* Categorical data were analyzed by the chi-squared test. ^*∗*^*P* < 0.05.

**Table 4:** Univariate and multivariate analyses of factors associated with OS.

Clinical and pathologic indexes		Univariate		Multivariate	
		HR (95% CI)	*P*	HR (95% CI)	*P*
Gender	(Male vs. female)	0.79 (0.51, 1.23)	0.30	N/A	N/A
Age, y	(≤50 vs. >50)	0.80 (0.60, 1.05)	0.11	N/A	N/A
HBsAg	(Positive vs. negative)	3.31 (2.49, 4.40)	0.00^*∗∗*^	1.52 (1.30, 1.78)	0.00^*∗∗*^
Liver cirrhosis	(No vs. yes)	0.79 (0.25, 2.48)	0.69	N/A	N/A
Portal lymph node	(Negative vs. positive)	0.54 (0.29, 1.03)	0.06	N/A	N/A
AFP (ng/ml)	(≤400 vs. >400)	2.33 (1.77, 3.06)	0.00^*∗∗*^	1.33 (1.15, 1.53)	0.00^*∗∗*^
MVI	(None vs. yes)	1.07 (0.79, 1.46)	0.65	N/A	N/A
Tumor number	(Multiple vs. single)	1.13 (0.83, 1.54)	0.45	N/A	N/A
Tumor size, cm	(≤5 vs. >5)	2.92 (2.16, 3.95)	0.00^*∗∗*^	1.51 (1.28, 1.79)	0.00^*∗∗*^
PVTT	(None vs. yes)	0.51 (0.39, 0.67)	0.00^*∗∗*^	0.99 (0.84, 1.16)	0.89
GGT (U/L)	(≤54 vs. >54)	1.48 (1.09, 2.01)	0.01^*∗*^	0.98 (0.83, 1.16)	0.85
Tumor encapsulation	(None vs. complete)	0.52 (0.39, 0.68)	0.00^*∗∗*^	0.82 (0.71, 0.96)	0.01^*∗*^
Edmondson stage	(II-IV vs. I-II)	0.87 (0.75, 1.00)	0.06	N/A	N/A
FOS	(Negative vs. positive)	0.69 (0.60, 0.79)	0.00^*∗∗*^	0.69 (0.60, 0.80)	0.00^*∗∗*^

AFP, *α*-fetoprotein; MVI, microvascular invasion; PVTT, portal vein tumor thrombus; GGT, *γ*-glutamyl transpeptidase; FOS, Fos protooncogene; HR, hazard ratio. Univariate analysis and multivariate analysis and cox proportional hazards regression model. ^*∗*^*P* < 0.05 and ^*∗∗*^*P* < 0.01

**Table 5:** Univariate and multivariate analyses of factors associated with TTR.

Clinical and pathologic indexes		Univariate		Multivariate	
		HR (95% CI)	*P*	HR (95% CI)	*P*
Gender	(Male vs. female)	0.99 (0.82, 1.20)	0.91	N/A	N/A
Age, y	(≤50 vs. >50)	0.87 (0.77, 1.00)	0.04^*∗*^	0.90 (0.78, 1.04)	0.15
HBsAg	(Positive vs. negative)	1.74 (1.52, 2.00)	0.00^*∗∗*^	1.64 (1.41, 1.91)	0.00^*∗∗*^
Liver cirrhosis	(No vs. yes)	0.75 (0.42, 1.33)	0.32	N/A	N/A
Portal lymph node	(Negative vs. positive)	0.66 (0.35, 1.24)	0.19	N/A	N/A
AFP (ng/ml)	(≤400 vs. >400)	1.30 (1.15, 1.48)	0.00^*∗∗*^	1.11 (0.96, 1.27)	0.16
MVI	(None vs. yes)	1.05 (0.91, 1.22)	0.50	N/A	N/A
Tumor number	(Multiple vs. single)	1.18 (1.03, 1.36)	0.02^*∗*^	1.35 (1.16, 1.57)	0.00^*∗∗*^
Tumor size, cm	(≤5 vs. >5)	1.52 (1.33, 1.74)	0.00^*∗∗*^	1.43 (1.23, 1.66)	0.00^*∗∗*^
PVTT	(None vs. yes)	0.75 (0.66, 0.86)	0.00^*∗∗*^	0.93 (0.80, 1.07)	0.30
GGT (U/L)	(≤54 vs. >54)	1.21 (1.05, 1.39)	0.01^*∗*^	1.01 (0.87, 1.19)	0.86
Tumor encapsulation	(None vs. complete)	0.80 (0.71, 0.91)	0.00^*∗∗*^	0.94 (0.82, 1.08)	0.37
Edmondson stage	(II-IV vs. I-II)	0.81 (0.71, 0.92)	0.00^*∗∗*^	0.81 (0.70, 0.93)	0.00^*∗∗*^
FOS	(Negative vs. positive)	0.79 (0.69, 0.90)	0.00^*∗∗*^	0.79 (0.68, 0.90)	0.00^*∗∗*^

AFP, *α*-fetoprotein; MVI, microvascular invasion; PVTT, portal vein tumor thrombus; GGT, *γ*-glutamyl transpeptidase; FOS, Fos protooncogene; HR, hazard ratio. Univariate analysis and multivariate analysis and cox proportional hazards regression model. ^*∗*^*P* < 0.05 and ^*∗∗*^*P* < 0.01.

## Data Availability

The data that support the findings of this study are available from the corresponding author upon reasonable request.

## Supplementary Materials

Supplementary Materials Supplementary Figure 1: examination of potential important pathways for liver carcinogenesis.
