# Supplementary figures and images for: Identification of FOS as a Candidate Risk Gene for Liver Cancer by Integrated Bioinformatic Analysis
Source: Biomed Res Int. 2020 Mar 22;2020:6784138. doi: 10.1155/2020/6784138 (PMC7125454; doi:10.1155/2020/6784138)

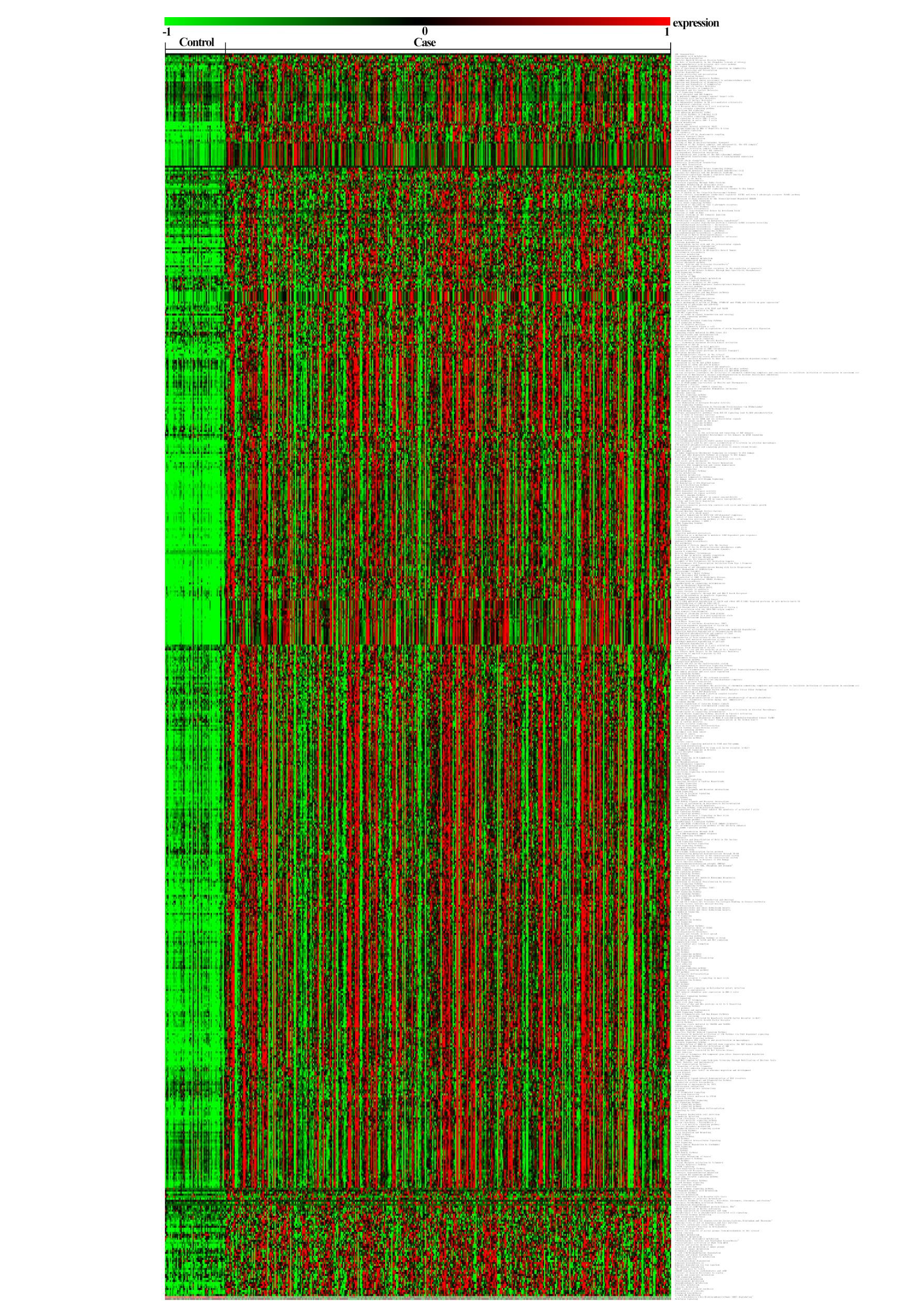

Supplement: Supplementary Materials — Supplementary Figure 1: examination of potential important pathways for liver carcinogenesis. [file 6784138.f1.tif]
